# Effect of zero-time exercise intervention on endometrial receptivity in women with thin endometrium a single-center randomized sham-controlled trial

**DOI:** 10.1007/s00404-026-08385-4

**Published:** 2026-03-16

**Authors:** Zhaohui Jiang, Yanjiao Hua, Li Deng, Yanmei Li, Shupei Xu, Mengjie Li, Yanfen Luo, Chunxia Wang, Gang Liu

**Affiliations:** Department of Reproductive Medicine, The Reproductive Hospital of Guangxi Zhuang Autonomous Region, Nanning, 530218 Guangxi Zhuang Autonomous Region China

**Keywords:** Physical activity, Intervention study, Assisted reproductive technology population, Sedentary lifestyle, Thin endometrium

## Abstract

**Background:**

Current therapeutic strategies for thin endometrium have inherent limitations, and zero-time exercise (ZTEx), a professionally guided fragmented low-intensity exercise, may serve as a novel adjunctive intervention for its management.

**Methods:**

This was a single-center randomized sham-controlled trial. Eligible patients undergoing ART were randomly divided into an experimental group (receiving ZTEx intervention) and a control group (receiving sham intervention). The ZTEx intervention was delivered over 12 weeks, featuring fragmented, low-intensity exercises under professional guidance. Key indicators of endometrial health—including endometrial thickness and endometrial receptivity-related parameters—were monitored throughout the intervention period.

**Results:**

After 12 weeks of intervention, ANCOVA (adjusted for baseline values) revealed that the experimental group had a significantly greater endometrial thickness than the control group (6.67 ± 1.15 mm vs. 5.88 ± 1.17 mm, *P* < 0.001). Concurrently, multiple endometrial receptivity-related indices improved significantly in the experimental group (all *P* < 0.05), including uterine artery hemodynamic parameters (pulsatility index [PI], resistance index [RI], systolic/diastolic ratio [S/D]) and vascularization indices (vascularization index [VI], flow index [FI], vascularization-flow index [VFI]).No exercise-related adverse events were reported over the course of the trial, confirming the safety profile of ZTEx.

**Conclusions:**

Supported by rigorous statistical analyses, ZTEx effectively enhances endometrial thickness and receptivity in ART patients with thin endometrium. Its key advantages—safety, feasibility, and no need for additional time or equipment—make it a promising adjuvant intervention in clinical ART settings.

**Supplementary Information:**

The online version contains supplementary material available at 10.1007/s00404-026-08385-4.

## What does this study add to the clinical work

**Table:** 

This single-center randomized sham-controlled trial finds that 12 weeks of professionally guided zero-time exercise may help increase endometrial thickness and improve several endometrial receptivity-related hemodynamic and vascularization parameters in ART patients with thin endometrium, and this low-intensity exercise modality with good safety and feasibility may serve as a potential non-pharmacological adjuvant option for clinical management of such patients in reproductive medicine practice.	

## Background

The endometrium is a critical site for embryonic implantation, and its thickness and receptivity exert a profound impact on pregnancy outcomes. According to compiled expert consensus and clinical guidelines, thin endometrium is defined as an endometrial thickness of ≤ 7 mm on the day dominant follicles reach maturity (diameter ≥ 18 mm) or on the day of human chorionic gonadotropin (HCG) administration [1]. With the delay in women’s reproductive age and the adjustment of fertility policies, the incidence of thin endometrium among the assisted reproductive technology (ART) population has shown an upward trend. Reported data indicate that its prevalence ranges from approximately 2.4 to 6.7%, with an even higher incidence observed in patients experiencing repeated implantation failure.

Currently, pharmacological interventions are the mainstay of clinical management for thin endometrium. Among these approaches, high-dose estrogen supplementation is the conventional first-line treatment. Nevertheless, approximately 50% of patients fail to achieve satisfactory therapeutic outcomes, and long-term administration of high-dose estrogen is associated with an increased risk of adverse events such as thromboembolism and breast disorder [2, 3]. Additionally, alternative modalities—including intrauterine perfusion of granulocyte colony-stimulating factor (G-CSF), platelet-rich plasma (PRP), pelvic floor neuromuscular electrical stimulation, and traditional Chinese medicine (TCM) therapy—have shown certain therapeutic benefits. However, these approaches are limited by multiple drawbacks, such as complex procedural requirements, high treatment costs, and inconsistent efficacy [4–7]. Thus, the development of a safe, convenient, cost-effective, and efficacious non-pharmacological intervention has emerged as an urgent unmet clinical need in the field of assisted reproduction.

Zero-time exercise (ZTEx), initially proposed by a research team from the University of Hong Kong, refers to a form of training that integrates simple strength and endurance exercises into fragmented intervals of daily life. It requires no additional equipment or dedicated time investment and can be performed anytime and anywhere. Working women are prone to pelvic blood circulation disorders and insulin resistance due to prolonged sedentary behavior, which in turn may lead to insufficient endometrial blood supply and reduced endometrial thickness [8–15]. Existing studies have confirmed that ZTEx effectively reduces sedentary time, improves insulin sensitivity, and enhances overall physical health, rendering it particularly suitable for time-constrained working populations.In contrast, moderate exercise can create favorable conditions for endometrial growth by improving pelvic circulation, regulating hormonal balance, and optimizing metabolic status [15–35]. Currently, research on exercise interventions in the field of assisted reproductive technology (ART) has primarily focused on high-intensity interval training (HIIT) or moderate-intensity aerobic exercise. However, these intervention modalities require dedicated time and venues, making them impractical for ART patients who frequently attend medical appointments and have sensitive physical conditions [20, 25, 33, 36].

As a low-intensity, fragmented exercise modality, ZTEx has not yet been reported for the treatment of thin endometrium. This study aimed to investigate the effects of ZTEx on endometrial thickness, endometrial receptivity-related indicators, and ART pregnancy outcomes in patients with thin endometrium via a single-center pseudo-randomized controlled trial, thereby providing a novel adjuvant therapeutic strategy for clinical practice.

## Methods

### Trial design and setting

**Fig. 1:**
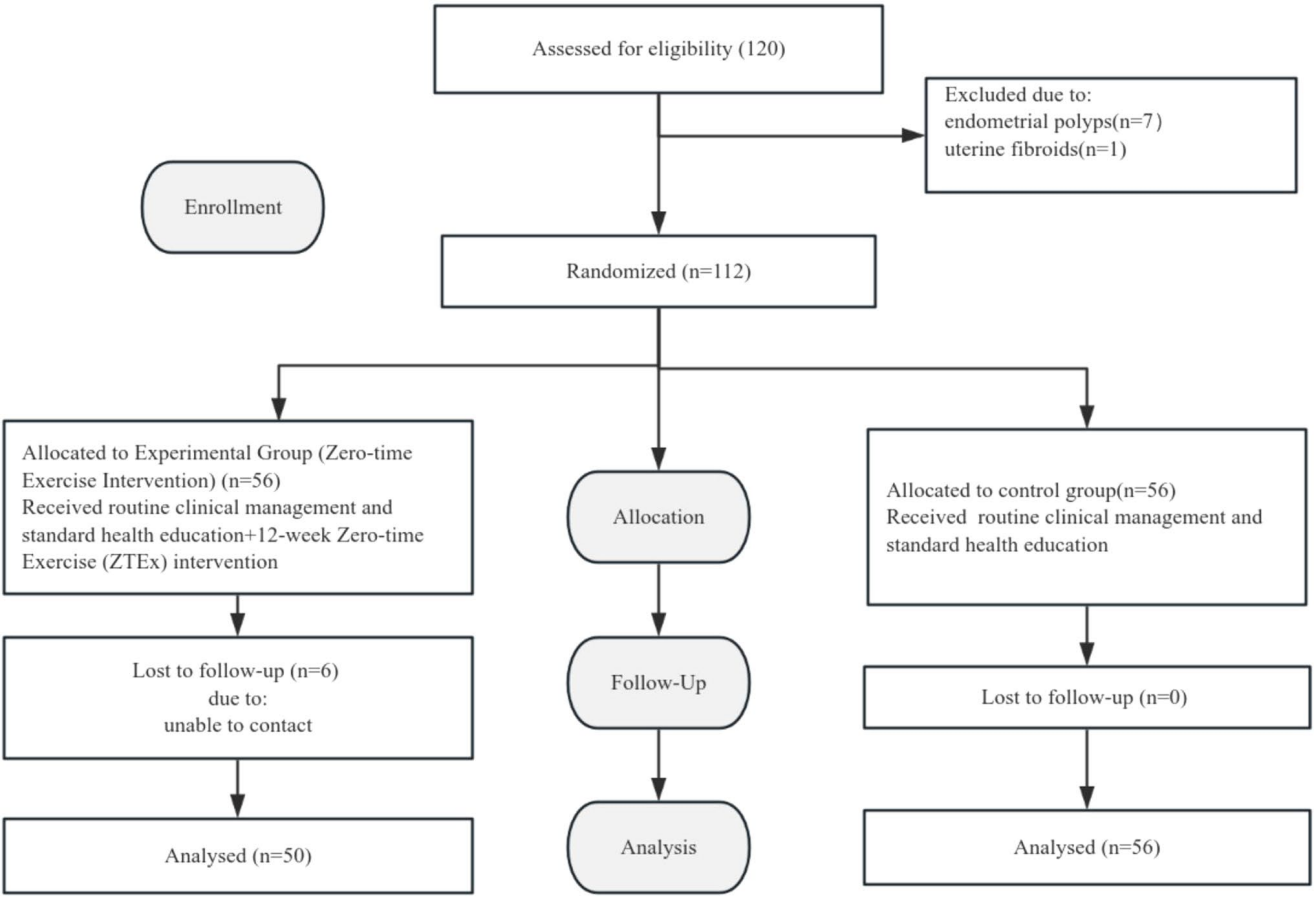
Flowchart of the study

This was a single-center, patient-blinded, randomized sham-controlled trial conducted at the Reproductive Hospital of Guangxi Zhuang Autonomous Region and reported in accordance with the CONSORT 2010 statement (Fig. 1). A complete CONSORT 2010 checklist, detailing compliance with each item, is provided in the Supplementary Materials.

### Study participants

A total of patients diagnosed with thin endometrium were prospectively recruited from the Reproductive Hospital of Guangxi Zhuang Autonomous Region between July 2024 and August 2025.

Inclusion Criteria:① Female patients aged 20–45 years with a confirmed desire for fertility; ② Endometrial thickness < 7 mm, as measured by transvaginal ultrasonography either in the mid-luteal phase of two consecutive menstrual cycles or on the day of human chorionic gonadotropin (HCG) injection; ③ Voluntary participation in the study with written informed consent provided.

Exclusion Criteria: ① Concurrent diagnosis of uterine organic pathologies, including uterine malformations, intrauterine adhesions, endometrial polyps, and uterine fibroids; ② Comorbidity with severe cardiovascular, hepatic, renal, endocrine, or autoimmune disorders; ③ Established contraindications to physical exercise; ④ Prior receipt of any interventions targeting endometrial thickening within 3 months before study enrollment; ⑤ Poor treatment compliance, which would preclude completion of the prescribed exercise intervention and scheduled follow-up assessments.

This study was approved by the Medical Ethics Committee of the Reproductive Hospital of Guangxi Zhuang Autonomous Region (Ethics Approval No.: KY-LL-2023-017). The China National Medical Research Classification Filing Information System is interconnected with the platform of the Chinese Clinical Trial Registry (ChiCTR). This study was registered in the China National Medical Research Classification Filing Information System (MR-45-25-014989).

### Sample size calculation

The primary outcome measure of this study was defined as the increment in endometrial thickness, and the sample size was calculated based on the findings of a prior systematic review and meta-analysis [33, 37]. Based on the distribution characteristics of standard deviations reported in the included studies, the effect size (i.e., the mean difference between the two groups) was set at 0.8 mm, with a pooled standard deviation of 1.2 mm. The sample size was estimated using the two-independent-samples *t* test, with the following preset parameters: a two-sided significance level (*α*) of 0.05, a statistical power (1-β) of 80%, and an equal 1:1 allocation ratio between the two groups. Using PASS 15.0 statistical software, the theoretical sample size was calculated as 34 participants per group.

To account for an anticipated dropout/loss-to-follow-up rate of 15%, the sample size was adjusted accordingly, with the final sample size set at 40 participants per group, resulting in a total of 80 participants for the study.

### Randomization and group allocation

To minimize the interference of the grouping procedure with patients’ clinical consultation needs, a 1:1 sequential allocation strategy was adopted based on the order of patients’ hospital visits. The detailed implementation procedures were as follows: ① Screening and Sequential numbering patients were registered and assigned consecutive natural numbers in real time by a dedicated researcher, with the sequencing based on the date and time of their first visit to the Department of Reproductive Medicine, Reproductive Hospital of Guangxi Zhuang Autonomous Region.

Eligibility confirmation included a definitive diagnosis of thin endometrium, fulfillment of all inclusion criteria, and provision of written informed consent; ② Group Assignment: using an alternating allocation rule, patients with odd-numbered identifiers were directly assigned to the experimental group, while those with even-numbered identifiers were allocated to the control group. Group assignment was performed synchronously by the same dedicated researcher responsible for registration and numbering, ensuring the smooth progression of patients’ clinical consultation workflow. A total of 50 patients were enrolled in the experimental group and 56 in the control group; ③ Group Confirmation and Documentation upon completion of group assignment, the allocation results immediately recorded in a dedicated recruitment registry by the responsible researcher. The registry was archived for future reference after all patients completed baseline indicator measurements.

### Intervention measures

Both the experimental and control groups received routine clinical management and standard health education, including advice on maintaining a balanced diet, regular sleep patterns, avoiding staying up late, and relieving psychological stress. On this basis, the experimental group received a 12-week Zero-time Exercise (ZTEx) intervention. The intervention protocol was optimized based on the findings of our preliminary studies [11, 14, 16–18, 21–24, 27, 29, 30, 32–43], as detailed in Supplementary Material 1. The specific intervention procedures were as follows: exercise Type fragmented, low-intensity exercises suitable for indoor performance were selected, with the specific modalities determined under the guidance of professional instructors.

Exercise intensity: the intensity was staged using the Rating of perceived exertion (RPE) scale: adaptation phase (Weeks 1–2): RPE 6–8; Enhancement phase (Weeks 3–4): RPE 9–12; Stabilization phase (Week 5 onwards): RPE 13–16. Concurrently, the target heart rate was recommended to reach 1.2–1.4 times the resting heart rate. An RPE intensity of 17–18 (defined as very strenuous) was reserved for female participants with a long-term regular exercise background. Exercise frequency and duration exercises were performed on ≥ 5 days per week. For every hour of sedentary behavior, participants were required to conduct 1–2 sessions of exercise, accumulating 10–15 min of total exercise time per session. Training management and supervision participants were provided with a dedicated exercise training kit, including instructional videos and illustrated movement guides. An exclusive WeChat group was established for daily exercise check-ins and real-time monitoring. Professional staff conducted weekly follow-up assessments and dynamically adjusted the intervention protocol based on each participant’s physical condition.

### Observation indicators and detection methods

Baseline data: baseline data were collected from patients in both groups, including age, body mass index (BMI), duration of infertility, infertility type (primary/secondary), pre-intervention endometrial thickness, mean menstrual cycle length (over 3 consecutive cycles), mean age at menarche, and mean menstruation duration (over 3 consecutive cycles).

Primary outcome measure: endometrial thickness was measured at the anterior, posterior, and lateral uterine walls before intervention and in the luteal phase 12 weeks post-intervention, with the average value calculated.

Secondary outcome measures: endometrial receptivity-related indicators were detected before intervention and in the luteal phase 12 weeks post-intervention, using the following methods:

Endometrial volume parameters: Three-dimensional (3D) ultrasound was used to obtain endometrial volume data, followed by calculation of the vascularization index (VI), flow index (FI), and vascularization-flow index (VFI). Blood flow-related indicators.

Subendometrial blood flow: Graded according to the Alder classification system (Grade 0: no blood flow; Grade I: minimal flow; Grade II: moderate flow; Grade III: abundant flow, including intraendometrial blood flow).Uterine artery hemodynamic parameters: The systolic/diastolic velocity ratio (S/D), pulsatility index (PI), and resistance index (RI) of the bilateral uterine arteries were measured. For each artery, measurements were taken over 3 consecutive cardiac cycles, and the average value was used. Other Receptivity Indicators: Endometrial pattern was classified based on the Gonen classification system (Type A, B, or C). Endometrial volume (V): Quantified via 3D ultrasound.Endometrial peristalsis: Observed using two-dimensional (2D) ultrasound and graded on a 4-point scale (Grade 0: no peristalsis; Grade 1: weak peristalsis; Grade 2: moderate peristalsis; Grade 3: strong peristalsis).

### Statistical analysis

Data analysis was performed using SPSS 26.0 statistical software. Continuous variables were expressed as mean ± standard deviation $(\overline{\mathrm{x}}\pm \mathrm{s})$, and categorical variables as frequencies and percentages (%). For intergroup comparisons of post-intervention continuous outcomes, Analysis of Covariance (ANCOVA) was used, with baseline values, age, and body mass index (BMI) included as covariates to adjust for potential confounding factors (consistent with the covariate adjustment criterion for causal inference). Intergroup comparisons of ordinal variables (subendometrial/intraendometrial blood flow pattern, endometrial peristalsis) were performed using the Mann–Whitney *U* test, while intragroup pre- and post-intervention comparisons were conducted using the paired t-test (for continuous variables) or the Wilcoxon signed-rank test (for ordinal variables). For multiple comparisons, Bonferroni correction was restricted to the primary outcome (endometrial thickness) to control for Type I error; secondary outcomes (VI, FI, VFI, etc.) were exploratory, with their P-values presented for reference without formal correction. A two-sided *P* value < 0.05 was considered statistically significant. Repeated measures ANOVA was further used to assess the time × group interaction effect.

## Results

### Comparison of baseline characteristics between the two groups

A total of 106 patients were enrolled in this study, with 50 allocated to the experimental group and 56 to the control group. No statistically significant differences were observed in baseline characteristics between the two groups, including age, body mass index (BMI), duration of infertility, infertility type, menstrual cycle length, and age at menarche (*P* > 0.05 for all comparisons), indicating good comparability between the two groups (Table 1).

**Table 1:** Comparison of baseline characteristics between groups

Parameters	Experimental group (zero-time exercise, *n* = 50)	Control group (*n* = 56)	Statistic (*t*/*χ*^2^)	*P* value
Age(years)	37.18 ± 4.39	37.73 ± 4.33	− 0.65	0.52
BMI (kg/m^2^)	22.82 ± 1.52	22.30 ± 1.69	1.66	0.1
Duration of infertility (years)	4.52 ± 1.07	4.61 ± 1.04	− 0.42	0.67
Menstrual cycle (days)	28.76 ± 2.49	29.20 ± 2.67	− 0.87	0.39
Age at menarche (years)	13.54 ± 1.23	13.30 ± 1.06	1.06	0.29
Type of infertility [*n* (%)]				
Primary infertility	6 (12.00%)	4 (7.14%)	0.73	0.39
Secondary infertility	44 (88.00%)	52 (92.86%)		

*BMI* body mass index

Prior to the intervention, no statistically significant difference was observed in endometrial thickness between the two groups (*P* > 0.05). After 12 weeks of intervention. Analysis of Covariance (ANCOVA), adjusted for baseline values, showed that the endometrial thickness in the experimental group (6.67 ± 1.15 mm) was significantly higher than that in the control group (5.88 ± 1.17 mm) (*t* = 3.53, *P* < 0.001).

**Fig. 2:**
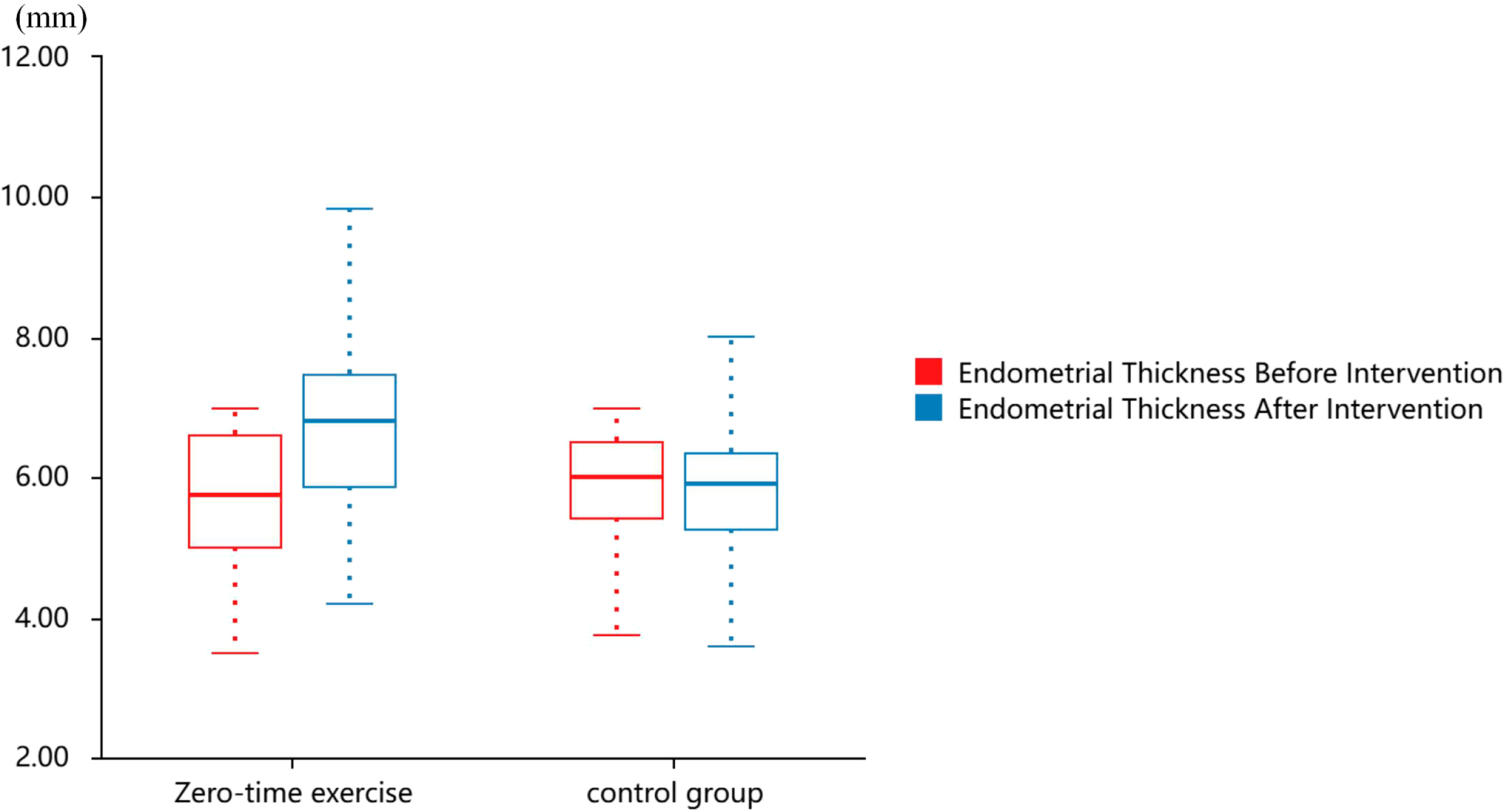
Comparison of endometrial thickness between groups before and after intervention

Intragroup comparisons using the paired *t* test revealed a significant increase in endometrial thickness in the experimental group compared with pre-intervention values (*P* < 0.001), while no significant change was observed in the control group (*P* > 0.05) (Table 2, Fig. 2).

**Table 2:** Comparison of endometrial thickness between groups before and after intervention

Time point	Zero-time exercise (*n* = 50, mean ± SD)	control group (*n* = 56, mean ± SD)	Statistical method	Test statistic (t for *t* test)	*P* value
Pre-intervention	5.698 ± 1.043	5.764 ± 0.970	^b	− 0.339	0.735
Post-intervention	6.674 ± 1.149	5.876 ± 1.172	^c	4.47703	< 0.001**
Intragroup (Pre → Post)	**–**	**–**	^a	− 7.155	< 0.001**/0.441

Multiple comparisons were adjusted using the Bonferroni correction to control for type I error

Intragroup *P* values are presented as zero-time exercise group/control group

*P < 0.05, **P < 0.01.

^a Paired t-test (continuous variables)

^b Independent samples *t* test

^c Analysis of covariance (ANCOVA): post-intervention comparisons adjusted for baseline values

Prior to the intervention, no statistically significant differences were found between the two groups in bilateral uterine blood flow-related indices (VI, FI, VFI, subendometrial/intraendometrial blood flow patterns), uterine artery hemodynamic parameters (PI, RI, S/D), blood flow volume, or endometrial peristalsis (all *P* > 0.05; Table 3).

**Table 3:** Comparisons of blood flow-related indices (VI, FI, VFI), uterine artery hemodynamic parameters (PI, RI, S/D), blood flow volume, and endometrial peristalsis

Outcome measure	Time point	Group		Statistical Method	Test Statistic (t for *t* test; *Z* for Wilcoxon; *U* for Mann–Whitney)	*P* value
		Zero-time exercise [*n* = 50, Mean ± SD, median (P25, P75)]	Control group [*n* = 56, Mean ± SD, median (P25, P75)]			
Continuous hemodynamic indices						
Vascularization index (VI, %)	Pre-intervention	0.436 ± 0.307	0.555 ± 0.195	^b	– 2.349	0.021*
	Post-intervention	6.125 ± 7.384	0.591 ± 0.128	^c	5.299	< 0.001**
	Intragroup (pre → post)	**–**	**–**	^a	– 5.496	< 0.001**/0.243
Flow index (FI)	Pre-intervention	0.069 ± 0.043	0.091 ± 0.055	^b	– 2.221	0.029*
	Post-intervention	16.443 ± 4.786	0.866 ± 0.041	^c	23.013	< 0.001**
	Intragroup (pre → post)	**–**	**–**	^a	– 24.216	< 0.001**/ < 0.001**
Vascularization-Flow index (VFI)	Pre-intervention	0.000 ± 0.000	0.000 ± 0.000	^b	– 1.188	0.238
	Post-intervention	1.089 ± 1.493	0.005 ± 0.001	^c	5.132	< 0.001**
	Intragroup (pre → post)	**–**	**–**	^a	– 5.155	< 0.001**/ < 0.001**
Pulsatility index (PI) of left uterine artery	Pre-intervention	3.097 ± 1.277	2.795 ± 1.309	^b	1.2	0.233
	Post-intervention	1.974 ± 0.411	2.494 ± 0.548	^c	– 5.541	< 0.001**
	Intragroup (pre → Post)	**–**	**–**	^a	5.932	< 0.001**/0.118
Resistance index (RI) of left uterine artery	Pre-intervention	0.909 ± 0.261	0.830 ± 0.214	^b	1.678	0.097
	Post-intervention	0.803 ± 0.059	0.862 ± 0.058	^c	– 5.173	< 0.001**
	Intragroup (pre → post)	**–**	**–**	^a	0.106	2.771/0.288
Pulsatility index (PI) of right uterine artery	Pre-intervention	2.175 ± 0.432	2.027 ± 0.387	^b	1.855	0.066
	Post-intervention	1.752 ± 0.615	2.013 ± 0.352	^c	– 2.643	0.010**/0.832
	Intragroup (pre → post)	**–**	**–**	^a	4.384	< 0.001**
Resistance index (RI) of right uterine artery	Pre-intervention	0.832 ± 0.061	0.914 ± 0.763	^b	– 0.758	0.45
	Post-intervention	0.714 ± 0.123	0.774 ± 0.063	^c	– 3.127	0.003**
	Intragroup (pre → post)	**–**	**–**	^a	7.779	< 0.001**/0.181
Systolic/diastolic ratio (S/D) of left uterine artery	Pre-intervention	5.731 ± 1.576	5.716 ± 1.316	^b	0.055	0.956
	Post-intervention	5.100 ± 1.436	5.630 ± 1.099	^c	– 2.113	0.037*
	Intragroup (pre → post)	**–**	**–**	^a	2.558	0.014*/0.642
Systolic/diastolic ratio (S/D) of right uterine artery	Pre-intervention	5.893 ± 0.681	5.608 ± 0.806	^b	1.954	0.053
	Post-intervention	4.616 ± 1.562	5.894 ± 1.107	^c	– 4.81	< 0.001**
	Intragroup (pre → post)	**–**	**–**	^a	5.5	< 0.001**/0.082
Blood flow volume (V, ml/min)	Pre-intervention	1.892 ± 0.565	2.032 ± 0.665	^b	– 1.163	0.247
	Post-intervention	2.885 ± 0.450	2.099 ± 0.294	^c	10.746	< 0.001**
	Intragroup (pre → post)	**–**	**–**	^a	– 12.349	< 0.001**/0.451
Ordinal endometrial blood flow and peristalsis indices						
Subendometrial blood flow (type I = 1; type II = 2; type III = 3)	Pre-intervention	2.000(2.000,2.000)	1.000(0.000,2.000)	^d	*U* = 955 *z* = – 3.018	0.003**
	Post-intervention	2.000(2.000,3.000)	1.000(1.000,2.000)	^d	*U* = 418.5 *z* = – 6.902	< 0.001**
	Intragroup (pre → post)	**–**	**–**	^a	*z* = – 4.481	< 0.001**
Intraendometrial blood flow pattern (type I = 1; Type II = 2; Type III = 3)	Pre-intervention	1.000(1.000,2.000)	1.000(1.000,2.000)	^d	*U* = 1350 *z* = – 0.358	0.72
	Post-intervention	2.000(1.000,3.000)	2.000(1.000,2.000)	^d	*U* = 1030.5 *z* = – 2.577	0.010**
	Intragroup (pre → post)	**–**	**–**	^a	*z* = – 3.639	< 0.001**
Endometrial peristalsis (grade 0-III)	Pre-intervention	0.000(0.000,1.000)	0.000(0.000,1.000)	^d	*U* = 1210.5 *z* = – 1.379	0.168
	Post-intervention	0.000(0.000,1.000)	0.000(0.000,1.000)	^d	*U* = 1391 *z* = – 0.072	0.943
	Intragroup (pre → post)	**–**	**–**	^a	*z* = 1.827	0.068

Intragroup *P* values are presented as zero-time exercise group/control group

Multiple comparisons were adjusted using the Bonferroni correction to control for type I error

*VI* vascularization index, *FI* flow index, *VFI* vascularization-flow index, *RI* resistance index, *S/D* systolic/diastolic ratio, *ANCOVA* analysis of covariance

*P < 0.05, **P < 0.01

^a Paired *t* test (continuous variables) / Wilcoxon signed-rank test (ordinal variables)

^b Independent samples *t* test

^c Analysis of covariance (ANCOVA): post-intervention comparisons adjusted for baseline values

^d Mann–Whitney *U* test

After 12 weeks of intervention, the experimental group showed significant improvements in all indicators except endometrial peristalsis (Table 3): ① Subendometrial/intraendometrial blood flow patterns (ordinal variables): the Mann–Whitney *U* test showed significant differences between groups (*P* < 0.01), with intra-group Wilcoxon signed-rank tests confirming improvements in the experimental group (*P* < 0.001); ② VI (1.14 ± 0.19 vs 0.59 ± 0.13), FI (17.69 ± 5.95 vs 14.54 ± 5.82), VFI (0.17 ± 0.09 vs 0.09 ± 0.06): ANCOVA showed significant inter-group differences (all *P* < 0.01); ③ Uterine artery PI, RI, S/D: these parameters were significantly lower in the experimental group (all *P* < 0.05 or *P* < 0.01); ④ Blood flow volume: this parameter was significantly higher in the experimental group (2.88 ± 0.45 vs 2.10 ± 0.29 mL/min, *P* < 0.001). No significant changes were observed in all indicators of the control group (all *P* > 0.05).

## Discussion

Although conventional pharmacological treatments can improve endometrial thickness in a subset of patients, they are associated with limited efficacy and potential safety concerns. Therefore, exploring non-pharmacological interventions is of considerable clinical significance.

The present study is the first to apply Zero-time Exercise (ZTEx) as an adjuvant therapy for women with thin endometrium. Our findings demonstrated that a 12-week ZTEx intervention significantly increased endometrial thickness and improved endometrial receptivity-related indices, thereby providing an effective therapeutic strategy for the management of thin endometrium.

Compared with other exercise modalities (e.g., HIIT), ZTEx has unique advantages: it requires no dedicated venue, additional time or equipment, which makes it highly acceptable and sustainable for time-constrained ART patients. We also supplemented adherence assurance measures (WeChat check-ins and weekly follow-ups) to further confirm its strong feasibility in clinical practice [11, 22, 33]. In the present study, the mean endometrial thickness of patients in the experimental group increased following 12 weeks of ZTEx intervention.

This finding is consistent with the established mechanism by which exercise enhances pelvic blood circulation. Specifically, ZTEx stimulates the contraction and relaxation of pelvic muscles through relevant movements, which in turn promotes uterine artery blood perfusion, augments oxygen and nutrient supply to the endometrium, and thus provides sufficient sustenance for endometrial cell proliferation [16, 17, 31, 35, 37, 42, 44]. Additionally, exercise can improve insulin sensitivity and mitigate the inhibitory effect of insulin resistance on endometrial growth [9, 14, 33, 40], which may represent one of the key mechanisms underlying ZTEx-induced endometrial thickening.

Endometrial receptivity is a critical determinant of successful embryo implantation, and ultrasound-derived parameters (e.g., hemodynamic indices, volumetric parameters, endometrial patterns, and peristaltic activity) are widely used and reliable tools for the clinical assessment of endometrial receptivity [45–48]. Specifically, decreased systolic/diastolic ratio (S/D), pulsatility index (PI), and resistance index (RI) of the uterine artery are indicative of improved uterine blood supply, whereas elevated vascularization index (VI), flow index (FI), and vascularization-flow index (VFI) reflect enhanced endometrial angiogenesis and blood perfusion.

We supplemented specific changes in receptivity indices (e.g., left uterine artery PI and VI values with corresponding *P* values) to clarify the link between index improvements and the optimized implantation microenvironment, while refining the description of the dual mechanisms, which confirms the comprehensive therapeutic effect of ZTEx. These findings suggest that ZTEx optimizes endometrial blood perfusion and ameliorates the endometrial implantation microenvironment. The potential mechanisms underlying these effects may be twofold: first, ZTEx directly stimulates the contraction and relaxation of pelvic muscles through professionally guided fragmented movements, thereby enhancing uterine artery blood perfusion and reducing vascular resistance [27, 29–31, 35, 40, 43]; second, exercise modulates the body’s endocrine homeostasis, creating a favorable microenvironment for endometrial angiogenesis and cell proliferation [18–20].

We emphasized the clinical significance of the absence of exercise-related adverse events throughout the trial, and supplemented ZTEx’s key characteristics (low cost and ease of implementation) to align with the clinical needs of ART patients, which fully confirms its high safety and strong applicability in clinical practice.

The present study has several limitations that should be acknowledged. First, the relatively small sample size and single-center design may have introduced bias, which limits the generalizability of the findings. Multi-center, large-sample randomized controlled trials are therefore warranted to validate these results in future research. Second, long-term follow-up of the participants was not performed, so the impact of ZTEx on pregnancy outcomes and offspring health remains unclear. Third, the molecular mechanisms underlying ZTEx-mediated endometrial growth were not explored in depth, and further basic experimental studies are required to elucidate these mechanisms.

In conclusion, ZTEx can effectively increase endometrial thickness and improve endometrial receptivity in women with thin endometrium. With the advantages of safety, feasibility and cost-effectiveness, ZTEx has the potential to serve as a routine adjuvant intervention for the management of thin endometrium.

## Conclusions

We supplemented specific endometrial thickness values before and after the intervention (with corresponding P values), clarified ZTEx’s key advantages (no additional time or equipment required, low cost, high safety), and added a perspective on future research verification (multi-center studies and long-term outcome assessments), which summarizes the core strengths of our study.

## Supplementary Information

Below is the link to the electronic supplementary material.

Supplementary file1 (XLSX 10 KB)

## Data Availability

All data generated or analyzed during this study are included in this published article.

## Abbreviations

ZTEx: Zero-time exercise
ART: Assisted reproductive technology
HCG: Human chorionic gonadotropin
BMI: Body mass index
PI: Pulsatility index
RI: Resistance index
S/D: Systolic/diastolic ratio
VI: Vascularization index
FI: Flow index
VFI: Vascularization-flow index
RPE: Rating of perceived exertion
G-CSF: Granulocyte colony-stimulating factor
PRP: Platelet-rich plasma
TCM: Traditional Chinese medicine
HIIT: High-intensity interval training
